# Familial Mediterranean fever in which Crohn’s disease was suspected: a case report

**DOI:** 10.1186/1756-0500-7-678

**Published:** 2014-09-27

**Authors:** Satohiro Matsumoto, Shunsuke Urayoshi, Yukio Yoshida

**Affiliations:** Department of Gastroenterology, Saitama Medical Center, Jichi Medical University, 1-847 Amanuma, Omiya, Saitama, Saitama, 330-8503 Japan

**Keywords:** Familial Mediterranean fever, Autoinflammatory disease, Colchicine, Crohn’s disease

## Abstract

**Background:**

Familial Mediterranean fever is a hereditary autoinflammatory disease, mainly characterized by periodic fever and serositis. The level of awareness about familial Mediterranean fever is far from sufficient, and it is assumed that there may be many patients with this disease who are under observation without an accurate diagnosis.

**Case presentation:**

A 30-year-old Japanese man presented to us with a few years’ history of recurrent episodes of fever, abdominal pain and diarrhea. He often visited a hospital when the attacks occurred; however, acute enteritis was diagnosed each time, and the symptoms resolved spontaneously within a few days. When he noticed a shortening of the interval between the attacks, he visited the hospital again. Upper endoscopy and colonoscopy performed at this hospital revealed no significant abnormal findings. He was then referred to our hospital under the suspicion of a small intestinal disease. Abdominal computed tomography revealed wall thickening and increased density of the mesenteric adipose tissue in the jejunum, which led us to suspect Crohn’s disease. Oral double-balloon enteroscopy was performed; because this revealed only mild mucosal edema in the jejunum, Crohn’s disease was considered to be highly improbable. Based on the patient’s clinical course, we suspected familial Mediterranean fever. As the Livneh criteria for familial Mediterranean fever were satisfied, the patient was started on oral colchicine for the purpose of diagnostic treatment. A definitive diagnosis of familial Mediterranean fever was then made based on the detection of a mutation of the Mediterranean fever gene. A marked reduction in the frequency of attacks was observed in response to colchicine treatment.

**Conclusions:**

Although Crohn’s disease may be considered first in the differential diagnosis of young patients presenting with periodic fever, abdominal pain and diarrhea, the possibility of familial Mediterranean fever should also be borne in mind.

## Background

Familial Mediterranean fever (FMF) is a hereditary periodic syndrome with autosomal recessive inheritance that is characterized by periodic fever and serositis, including peritonitis and pleuritis. FMF was first reported in 1908 by Janeway and Mosenthal [1]. It is most frequently encountered among the hereditary periodic syndromes and is estimated to affect 100,000 or more patients in the world [2]. Abdominal pain and fever are the most frequent clinical symptoms, which often makes definitive diagnosis difficult. Herein, we report our experience of a case of FMF in which a few years elapsed before a definitive diagnosis could be made, with the patient initially suspected as having Crohn’s disease (CD).

## Case presentation

A 30-year-old Japanese man presented with a few years’ history of recurrent episodes of fever, abdominal pain and diarrhea that lasted for a few days and occurred a few times a year. At the time of an attack, he experienced abdominal pain spreading from the periumbilical area to the entire abdomen, a few episodes of watery diarrhea per day, and fever of approximately 38°C, all of which were alleviated within a few days by fasting and resting. He often visited a medical institution when the attacks occurred; however, each time, he was diagnosed as having acute enteritis. Between attacks, the patient was asymptomatic and had normal stools. When he noticed that the attacks had occurred once a month for 3 months in a row, he visited the medical institution again. Upper and lower gastrointestinal endoscopy performed at the institution did not reveal any significant abnormal findings. The patient was then referred to our hospital under the suspicion of a small intestinal disease.

**Figure 1:**
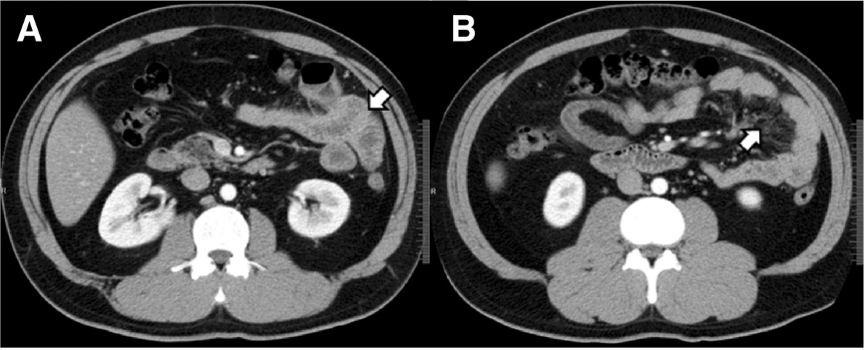
**Findings of abdominal computed tomography (CT). A**. showing a near-circumferential wall thickening in the jejunum (arrow). **B**. showing increased density of the mesenteric adipose tissue in the jejunum (arrow).

**Figure 2:**
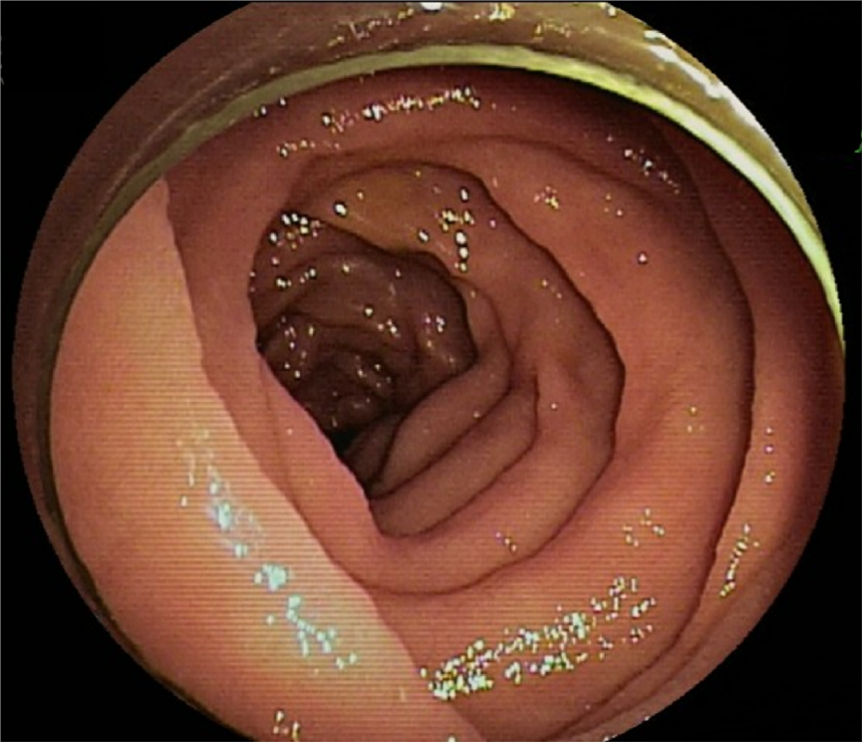
**Findings of double-balloon enteroscopy (DBE).** Oral DBE reveals mild mucosal edema in the upper segment of the jejunum.

At the first visit, the patient presented with a fever of approximately 38°C, abdominal pain, and a few episodes of watery diarrhea per day. As the results of the blood test at this visit, the findings of a white blood cell (WBC) count of 8,060/μL (neutrophil [Neut]: 72.1%), serum C-reactive protein (CRP) level of 14.64 mg/dL, and erythrocyte sedimentation rate (ESR) of 39 mm/hr suggested the presence of inflammation. He was admitted for detailed examination and treatment 1 week later. On admission, there was no fever, and the abdominal pain and diarrhea had resolved. A repeat blood test revealed a WBC count of 5,370/μL (Neut: 53.3%), serum CRP of 4.69 mg/dL, and ESR of 29 mm/hr, showing a trend towards reduction of the inflammatory markers. Thyroid function tests and serological tests for various autoantibodies also revealed no abnormal findings.As abdominal computed tomography (CT) revealed wall thickening and increased density of the mesenteric adipose tissue in the upper small intestine (Figure 1), we suspected CD and performed oral double-balloon enteroscopy (DBE). Although mild mucosal edema was detected in the jejunum, neither erosions nor ulcers were observed on the mucosal surface (Figure 2). A biopsy specimen obtained from the edematous mucosa of the jejunum revealed partial and slight inflammatory cell infiltration and edematous changes. As a subsequently performed transanal DBE revealed no abnormal findings, CD was considered to be highly improbable.

We suspected FMF based on the clinical course and evaluated the Livneh criteria for FMF (Table 1) [3]. Because the criteria were satisfied, oral administration of colchicine was initiated at a daily dose of 0.5 mg. Molecular analysis for FMF was done by sequencing of exon 1 and exons of 3–10 of familial Mediterranean fever (*MEFV*) gene. The results of genetic analysis for the *MEFV* gene demonstrated a heterozygous mutation of M694I in exon 10. A definitive diagnosis of FMF was then made. The patient did not have any family history of the disease. Subsequently, although the attacks were not completely eliminated, their frequency markedly decreased. Only loose stools occurred for 2 to 3 days every 1 to 2 months, but there was no fever, abdominal pain, or increases of the inflammatory markers in the blood test. In order to achieve remission of the symptoms, an increase in the dose of colchicine was considered. However, because mild hepatic function impairment possibly caused by colchicine was noted, the treatment has been maintained at the initial dose. Approximately 1 year has elapsed since the initiation of the colchicine therapy, and the disease state remains stable.

**Table 1:** **Diagnostic criteria for familial Mediterranean fever** [3]

*Major criteria*: typical attack*	*Minor criteria*
1. Peritonitis (generalized)	1. Incomplete attacks affecting one or more sites
2. Pleurisy (unilateral) or pericarditis	-Abdomen
3. Monoarthritis (hip, knee, ankle)	-Lungs
4. Isolated fever	-Joints
*axillary temperature >38°C, duration 12–72 h and ≥ three attacks	2. Exertion-related leg pain
	3. Response to colchicine

The diagnosis requires at least one major criteria or at least two minor criteria.

## Discussion

FMF is a disease with a high prevalence rate in the Mediterranean basin and is considered to be associated with the highest morbidity among the hereditary periodic syndromes [2]. In Japan, the first case of FMF was reported by Hayashi *et al*. in 1976 [4]. An epidemiologic study conducted in 2009 estimated the total number of patients with the disease in Japan at 292 [5]. The level of awareness about this disease is far from sufficient, and it is assumed that there may be many patients with this disease who are under observation without an accurate diagnosis.

Although the age at onset is considered to be less than 20 years in 90% of the cases, it is 19.6 ± 15.3 years in Japan, which is higher than that reported from overseas. Furthermore, it has been reported that in 37.3% of the Japanese cases, the disease onset occurs at the age of 20 years or later [5]. Typically, an attack starts suddenly, mainly with fever lasting for 1 to 4 days (6 to 96 hours), and abdominal pain observed in 95% or more of the cases. Diarrhea, which was observed in our patient, has been reported to occur in 10% to 20% of the patients [2]. In our patient, who presented with recurrent abdominal pain and diarrhea, small bowel CD was suspected at first, because abdominal CT revealed thickening of the upper small intestinal wall. Although blood tests revealed an increase in the levels of inflammatory markers, there was no weight loss, hypoalbuminemia, hypocholesterolemia, iron-deficiency anemia, etc. CD was definitively ruled out by DBE. On the other hand, there are reports on concurrent occurrence of FMF and CD [6–8], and also the beneficial effect of colchicine for CD associated with FMF [8].

The M694I mutation was the one most frequently found in Japanese patients with FMF. The majority of the patients were compound heterozygous or complex allele for M694I mutations. The 17.5% of *MEFV* mutations pattern were M694I alone [9]. FMF is basically an autosomal recessive disease; therefore, double mutations cause the disease. However, the genotype of some families showed autosomal dominant inheritance of FMF [10].

Colchicine is considered to be the agent of first choice for FMF, as it is highly effective [11–14]. Furthermore, it can prevent the onset of amyloidosis, which is a complication that greatly affects the outcome, and can arrest the progression of amyloidosis even after the onset. Colchicine is also effective for the prevention and treatment of renal amyloidosis [11, 15].

There are few reports on the endoscopic and pathological findings of FMF. In our patient, because we found a localized lesion on abdominal CT and determined that detailed pathological examination would be required, DBE was performed instead of double-contrast examination of the small intestine. DBE only revealed mild edematous change of the small intestinal mucosal surface. Because FMF is primarily characterized by peritonitis, there may be no abnormal findings or only mild abnormalities on the mucosal surface.

Since the most common chief complaints are fever and abdominal pain, the clinical presentation of FMF is often difficult to differentiate from that of acute abdomen. Thus, it is assumed that there may be patients with FMF who unnecessarily undergo appendectomy or cholecystectomy. Because the features of Behcet’s disease also sometimes satisfy the diagnostic criteria of FMF, careful diagnosis is required [9]. It is expected that many patients with FMF visit outpatient gastrointestinal clinics. In our patient, a few years had elapsed before a definitive diagnosis could be made.

## Conclusions

Although our patient was initially suspected to have CD, the disease was excluded by DBE. When patients with periodic fever or peritonitis of unknown etiology are encountered, FMF should be included in the differential diagnosis. Dissemination of information about FMF to gastroenterologists is also important.

### Consent

Written informed consent was obtained from the patient for publication of this Case Report and the accompanying images. A copy of the written consent is available for review by the Editor-in-Chief of this journal.

### Ethical consideration

We obtained the patient’s agreement to the genetic testing, but we didn’t receive ethics approval for the genetic testing.

## Abbreviations

FMF: Familial Mediterranean fever
CD: Crohn’s disease
WBC: White blood cell
Neut: Neutrophil
CRP: C-reactive protein
ESR: Erythrocyte sedimentation rate
CT: Computed tomography
DBE: Double-balloon enteroscopy.
